# Transcriptomics analyses of soybean leaf and root samples during water-deficit

**DOI:** 10.1016/j.gdata.2015.05.036

**Published:** 2015-06-04

**Authors:** Prateek Tripathi, Roel C. Rabara, Qingxi J. Shen, Paul J. Rushton

**Affiliations:** a Molecular and Computational Biology Section, University of Southern California, Los Angeles, CA 90089, USA; b Texas A&M AgriLife Research & Extension Center Dallas, TX 75252, USA; c School of Life Sciences, University of Nevada, Las Vegas, NV 89154, USA

**Keywords:** Transcriptomics, Drought, Microarray, Soybean, Genomics, WRKY

## Abstract

Drought being a major challenge for crop productivity and yield affects multigenic and quantitative traits. It is also well documented that water stress shows a cross talk with other abiotic stresses such as high temperature and high light intensities (Tripathi et al., 2013) [1]. In this report, we documented the details of the methods and quality controls used and considered in our time course-based transcriptome profile of soybean plants under water deficit conditions using microarray technology. The findings of this study are recently published by the Rushton lab in BMC Genomics for a comparative study of tobacco and Soybean (Rabara et al., 2015) [2]. The raw microarray data set is deposited in GEO database with accession number GSE49537.

**Table:** 

Specifications	
Organism/cell line/tissue	Soybean (*Glycine max*.), Four weeks old plants
Sex	NA
Sequencer or array type	Nimblegen custom based Microarray NimbleGen *Glycine max* Array [100526_Brach_MoGene_exp]
Data format	Raw Data
Experimental factors	Dehydrated and Un-dehydrated time course samples (roots and shoots)
Experimental features	Four week old hydrophonically grown plants were transferred to empty boxes (without touching the plants) and samples (roots and shoots separately) were collected at 0 h (control), 30 min, 1 h, 2 h, 3 h and 5 h and frozen immediately in liquid nitrogen for further processing.
Consent	NA
Sample source location	Brookings, South Dakota, USA

## 1 Direct link to deposited data

Deposited dataset can be found here : http://www.ncbi.nlm.nih.gov/geo/query/acc.cgi?acc=GSE49537.

## 2 Experimental design, materials and methods

### 2.1 Plant material and growth conditions

**Fig. 1:**
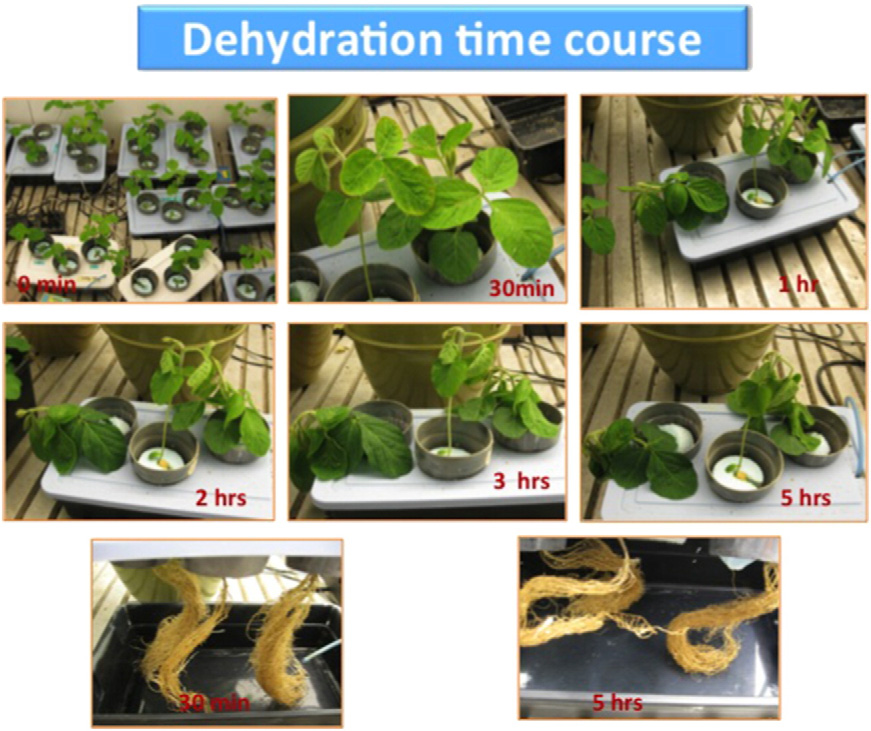
Schematics of the experimental set-up.

Soybean (Glycine max *L.*) W-82 seeds were soaked in water for 10 min and viable seeds were used for sowing. The seeds were sowed on a vermiculite–perlite mix (1:1) and after 2 weeks of growth plantlets were transferred to a hydroponics set-up with 0.5 × Hogland solution, pH 5.8 in a growth chamber (Conviron^R^) with a 16 h/8 h day/night cycle at 25 °C and 50% RH. The tissues (leaf and root) were harvested after 30 days of total growth when the second tri-foliate becomes fully visible. Plants were allowed to dehydrate in the growth chamber by transferring them to empty boxes for 6 time points (0 min, 30 min, 1 h, 2 h, 3 h and 5 h) of dehydration (Fig. 1) and harvested without actually touching the plants to nullify any possibility of wounding. Nine independent plants were utilized (three replicates per time point and three plants per replicate) for the study and after harvesting were immediately stored in − 80 °C. These samples were utilized for the transcriptomics purposes.

### 2.2 Sample collection and RNA preparation

An equal amount of tissue from each nine plants per time-point was pooled for RNA isolation. Tissue was homogenized using ceramic mortar and pestle in liquid nitrogen and RNA was isolated from both the tissues using QIAGEN© RNeasy-MIDI kit as per the user manual instructions. To get rid off the possible DNase contamination, DNase treatment was performed using Ambion's TURBO DNA-*free*™ Kit.

### 2.3 RNA quantification and quality check

Quality of the total RNA was checked using Nanodrop and AGILENT© Bioanalyzer-2100 using RNA600 chip as per the user manual instruction. 10 μg of total RNA from each tissue per time-point was used for microarray analysis. Our samples had high quality RNA as per the microarray analysis standards. Any sample with RNA Integrity Number (RIN) less than 7.0 and 260/230 ratio less than 1.7 were not used for the microarray analysis (Table 1).

### 2.4 Microarray data and data analysis

Microarray analyses was performed using a custom based 12 × oligo chip designed by NIBMELGEN, which constitutes 60mer of each high and low confidence gene from GLYMAv1.0 of soybean genome release from phytozome [3] along with manually curated 179 soybean *WRKY* genes obtained using bio-informatics pipeline described in Rushton et al. [4] and Tripathi et al. [5]. Oligoarray experiments were performed for 36 samples (18/tissue) at MOGENE, LC (St. Louis, MO). Data analysis was performed using *ArrayStar* v4 software package from DNASTAR (DNASTAR Inc., Madison, WI, USA). Differential regulation was calculated with FDR correction at 5%.

## 3 Discussion

In this briefing, we described a unique and a robust dataset of trancriptomic analyses of roots and leaf samples of soybean under water stress. This experimental set-up leads us to find some novel candidates, which provide many novel insights into the roles of WRKY transcription factors during water stress in soybean towards system-wide understanding of water-stress signaling as also discussed in Tripathi et al. [1]. This dataset has been recently used in a comparative study of soybean and tobacco under drought conditions [2]. We hope the design and dataset may also be useful for the different groups and investigations related to other transcription factors for crop improvement under drought conditions.

## Conflict of interest

The authors declare no conflicts if interest.

## Figures and Tables

**Table 1:** Concentration and RIN values of total RNA isolated from Soybean shoot & root samples.

S. no	Sample ID	Total RNA (μg)	RNA integrity number (RIN)
*Leaves*			
1	0 min 1	213.9	8.1
2	0 min 2	151.5	8.4
3	0 min 3	141.9	8.4
4	30 min 1	87.15	9.1
5	30 min 2	128.7	9.5
6	30 min 3	56.25	9.2
7	1 h 1	120.3	9.3
8	1 h 2	73.2	8.9
9	1 h 3	148.05	8.4
10	2 h 1	70	8.6
11	2 h 2	59.4	8.4
12	2 h 3	140.25	8.6
13	3 h 1	55.65	7.9
14	3 h 2	60.75	9.1
15	3 h 3	35.7	8.5
16	5 h 1	35	8.9
17	5 h 2	98.7	8.8
18	5 h 3	67	8

*Roots*			
1	0 min 1	66.45	8.6
2	0 min2	73.35	8.1
3	0 min 3	63.15	8.8
4	30 min 1	91.35	9.1
5	30 min 2	69.15	9.4
6	30 min 3	87.15	9
7	1 h 1	122.85	8.9
8	1 h 2	101.7	8.8
9	1 h 3	100.8	8.7
10	2 h 1	93.9	8.7
11	2 h 2	96.15	7.8
12	2 h 3	107.85	8.6
13	3 h 1	88.8	8.4
14	3 h 2	49.05	8.7
15	3 h 3	49	8.4
16	5 h 1	31.2	8.4
17	5 h 2	82.95	8.1
18	5 h 3	45.3	8.4
